# PP2A Mediated AMPK Inhibition Promotes HSP70 Expression in Heat Shock Response

**DOI:** 10.1371/journal.pone.0013096

**Published:** 2010-10-01

**Authors:** Ting Wang, Qiujing Yu, Juan Chen, Bo Deng, Lihua Qian, Yingying Le

**Affiliations:** Key Laboratory of Nutrition and Metabolism, Institute for Nutritional Sciences, Shanghai Institutes for Biological Sciences, Chinese Academy of Sciences and Graduate School of the Chinese Academy of Sciences, Shanghai, China; Texas A&M University, United States of America

## Abstract

**Background:**

Under stress, AMP-activated protein kinase (AMPK) plays a central role in energy balance, and the heat shock response is a protective mechanism for cell survival. The relationship between AMPK activity and heat shock protein (HSP) expression under stress is unclear.

**Methodology/Principal Findings:**

We found that heat stress induced dephosphorylation of AMPKα subunit (AMPKα) in various cell types from human and rodent. In HepG2 cells, the dephosphorylation of AMPKα under heat stress in turn caused dephosphorylation of acetyl-CoA carboxylase and upregulation of phosphoenolpyruvate carboxykinase, two downstream targets of AMPK, confirming the inhibition of AMPK activity by heat stress. Treatment of HepG2 cells with phosphatase 2A (PP2A) inhibitor okadaic acid or inhibition of PP2A expression by RNA interference efficiently reversed heat stress-induced AMPKα dephosphorylation, suggesting that heat stress inhibited AMPK through activation of PP2A. Heat stress- and other HSP inducer (CdCl_2_, celastrol, MG132)-induced HSP70 expression could be inhibited by AICAR, an AMPK specific activator. Inhibition of AMPKα expression by RNA interference reversed the inhibitory effect of AICAR on HSP70 expression under heat stress. These results indicate that AMPK inhibition under stress contribute to HSP70 expression. Mechanistic studies showed that activation of AMPK by AICAR had no effect on heat stress-induced HSF1 nuclear translocation, phosphorylation and binding with heat response element in the promoter region of HSP70 gene, but significantly decreased HSP70 mRNA stability.

**Conclusions/Significance:**

These results demonstrate that during heat shock response, PP2A mediated AMPK inhibition upregulates HSP70 expression at least partially through stabilizing its mRNA, which suggests a novel mechanism for HSP induction under stress.

## Introduction

Mammalian AMP-activated protein kinase (AMPK) is a heterotrimeric complex consisting of α, β, and γ subunits. AMPK is activated allosterically by an increase in the intracellular AMP/ATP ratios and/or by the phosphorylation of threonine 172 in the α subunit [1]. AMPK is initially viewed as a ‘fuel gauge’ to monitor cellular energy status in response to nutritional environmental variations. Physiological or pathological stimuli that deplete cellular energy levels such as prolonged exercise, metabolic poisoning, oxidative stress, hypoxia, ischemia, or nutrient deprivation, result in an increased AMP/ATP ratio that in turn activates AMPK [1]. The activation of AMPK coordinates a cellular program that limits further ATP depletion and promotes compensatory changes that maintain cellular ATP levels. Recent studies revealed that AMPK can also be activated by various stimuli independent of AMP/ATP ratio [2]. AMPK also plays important roles in cell survival and growth [3], [4].

Heat shock response is a universal protective mechanism for cell survival under various environmental and physiological stresses. A family of molecular chaperones, named heat shock proteins (HSPs), is dramatically increased in response to these stresses. HSPs are involved in protein folding, assembly, degradation, intracellular localization, etc. They are classified into families, and among them the HSP70 family appears to be the most evolutionarily conserved and distributed in animals [5]. HSP70 transcription in response to heat stress is mainly mediated by binding of heat shock transcription factor 1 (HSF1) to heat shock elements (HSE) in the promoter region of HSP70 gene. Before binding with HSE, HSF1 undergoes phosphorylation, trimerlization and nuclear translocation upon heat stress [6], [7]. However, the signaling pathway(s) involved in HSP expression in response to heat stress is unclear. Corton et al. [8] reported that exposed rat primary hepatocytes to heat stress significantly activated AMPK. However, Kodiha et al. [9] reported that heat stress inhibited AMPK in both Hela and 293 cells. Recently, Jung et al. [10] reported that activation of AMPK significantly inhibited HSP70 expression in Hela cells. In the present study, we first examined the effect of heat stress on AMPK activity in various cell types, then investigated the involvement of AMPK in HSP70 expression in response to heat stress and other stresses in HepG2 cells and explored the underlying mechanisms.

## Results

### AMPK is inhibited by phosphatase 2A (PP2A) upon heat stress

**Figure 1:**
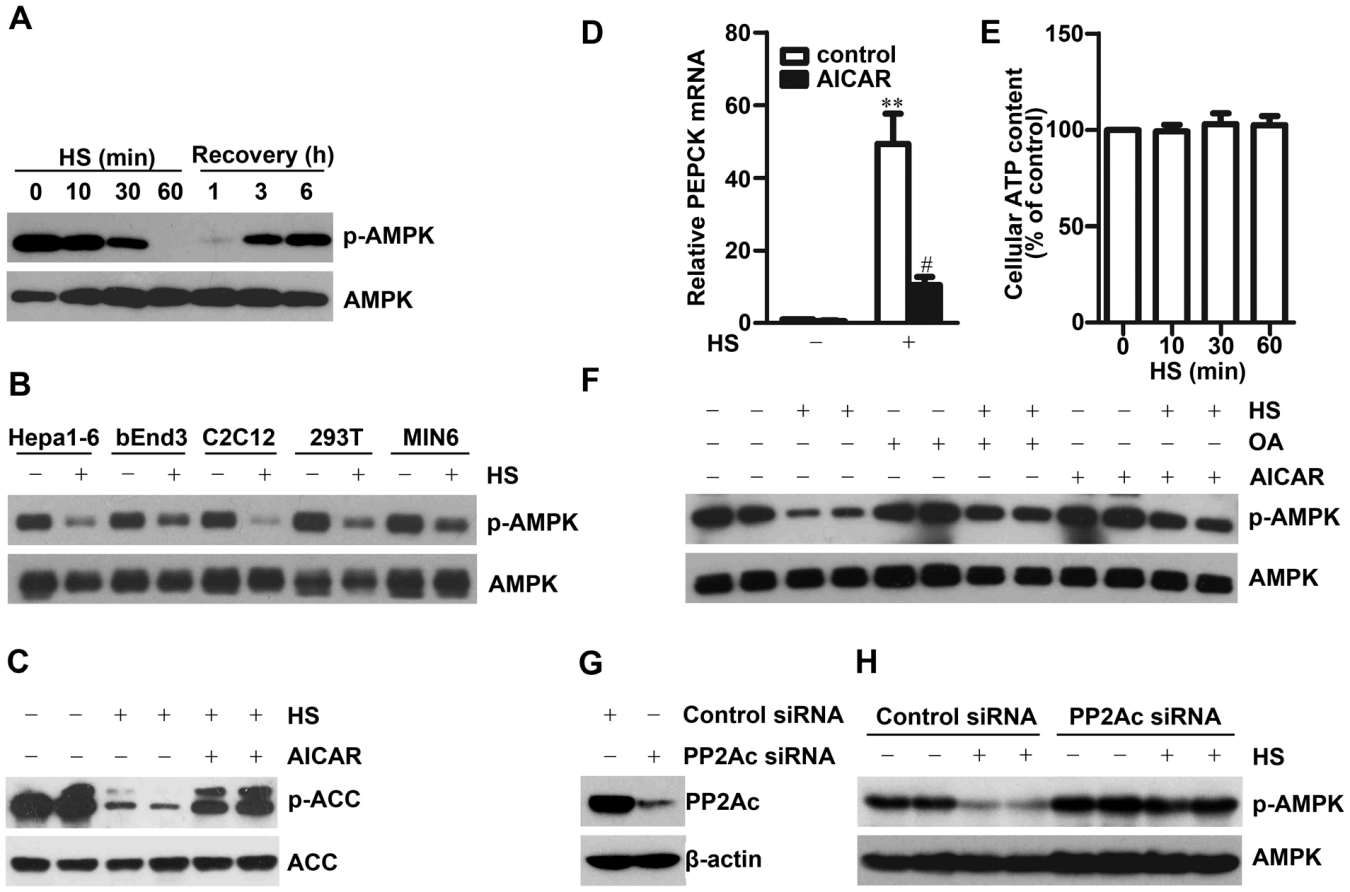
Heat stress induces AMPK dephosphorylation through activation of PP2A. **A.** HepG2 cells were exposed to 42°C (HS) for 0–1 h or 1 h followed by recovery at 37°C for 1–6 h. **B.** Various types of cells were exposed to 42°C (HS) for 30 min, then examined for AMPKα phosphorylation by Western blot. **C–D.** HepG2 cells pretreated with or without 1 mM AICAR for 15 min were exposed to 42°C (HS) for 30 min (**C**) or 1 h (**D**), then examined for ACC phosphorylation by Western blot (**C**), or PEPCK expression by real-time PCR (**D**). mean ± SEM, n = 3. **P<0.01 vs. cells without heat stress; ^#^P<0.05 vs. cells cultured in control medium under heat stress. **E.** HepG2 cells were exposed to 42°C (HS) for indicated times and measured intracellular ATP levels. mean ± SEM, n = 3. **F.** HepG2 cells pretreated with 100 nM okadaic acid (OA) for 30 min or 1 mM AICAR for 15 min were exposed to 42°C (HS) for 30 min, then examined for AMPKα phosphorylation by Western blot. **G–H.** bEend.3 cells transfected with PP2Ac specific siRNA or control siRNA for 48 h were exposed to 42°C (HS) for 30 min. PP2Ac protein level (G) and AMPKα phosphorylation (H) were examined by Western blot. **A–C and F**–**H:** experiments were performed at least three times and the representative results are shown.

We first examined the effect of heat stress on AMPK activity in HepG2 cells by Western blot with antibodies that recognize phosphorylated Thr172 in AMPKα subunit (AMPKα). As shown in Figure 1A, exposure of HepG2 cells to 42°C induced rapid dephosphorylation of AMPKα, and the phosphorylated AMPKα, was almost undetectable at 1 h after heat stress. AMPKα phosphorylation gradually recovered after the cells were switched to 37°C. These results were consistent with the results from Hela and 293 cells [9]. We also examined the effect of heat stress on AMPKα phosphorylation in other cell types, including Hepa1-6, bEnd.3, C2C12, 293T and MIN6. Heat stress resulted in dephosphorylation of AMPKα in all these cell types (Figure 1B), suggesting that AMPK inhibition is a general event in cells under heat stress.

As acetyl-CoA carboxylase (ACC) is a substrate for AMPK and phosphoenolpyruvate carboxykinase (PEPCK) gene expression is suppressed by AMPK activation, the phosphorylation of ACC and mRNA level of PEPCK serve as indicators of AMPK activity. While exposure of HepG2 cells to 42°C for 30 min significantly induced ACC dephosphorylation (Figure 1C) and PEPCK mRNA expression (Figure 1D), pretreatment of the cells with AICAR (5′ -aminoimidazole-4-carboxamide ribonucleoside), an AMPK specific activator, reversed heat stress-induced dephosphorylation of ACC (Figure 1C) and upregulation of PEPCK expression (Figure 1D). These results suggest that the inhibition of AMPK activity under heat stress in turn causes ACC activation and PEPCK upregulation.

As the activity of AMPK is modulated by intracellular AMP/ATP ratio, we examined the ATP levels in HepG2 cells after exposure to 42°C for different time periods and found that heat stress for up to 4 h had no significant effect on intracellular level of ATP (Figure 1E and data not shown). This result suggests that the rapid dephosphorylation of AMPK under heat stress may result from other mechanism(s) than ATP change.

It has been reported that PP2A could regulate the interaction between AMPKα2 and γ1 subunits [11], and dephosphorylate AMPKα in cell-free assays [12]. Recently, PP2A was reported to mediate glucose-, palmitate-, or ethanol-induced AMPK inhibition [13]–[15]. To determine whether the inhibitory effect of heat stress on AMPK is mediated by activation of PP2A, we treated HepG2 cells with 100 nM okadaic acid (OA), a cell-permeable inhibitor of PP2A, and found that OA efficiently reversed heat stress-induced dephosphorylation of AMPKα (Figure 1F). As a positive control, AICAR also reversed heat stress –induced AMPKα dephosphorylation. Okadaic acid can also inhibit PP1, but it inhibits PP1 activity with higher concentration [16]. 100 nM okadaic acid has been reported to inhibit PP2A but not PP1 [16], [17]. Therefore, our results suggest that PP2A may inhibit AMPK upon heat stress. To further confirm that heat stress inhibits AMPK through activation of PP2A, we inhibited PP2Ac expression by RNA interference (Figure 1G). Transfection with PP2Ac siRNA but not control siRNA restored the phosphorylation level of AMPK after the cells were exposed to heat stress (Figure 1H), supporting that heat stress-induced AMPK inhibition is due to the activation of PP2Ac.

### AMPK inhibition under stress contributes to HSP70 upregulation

**Figure 2:**
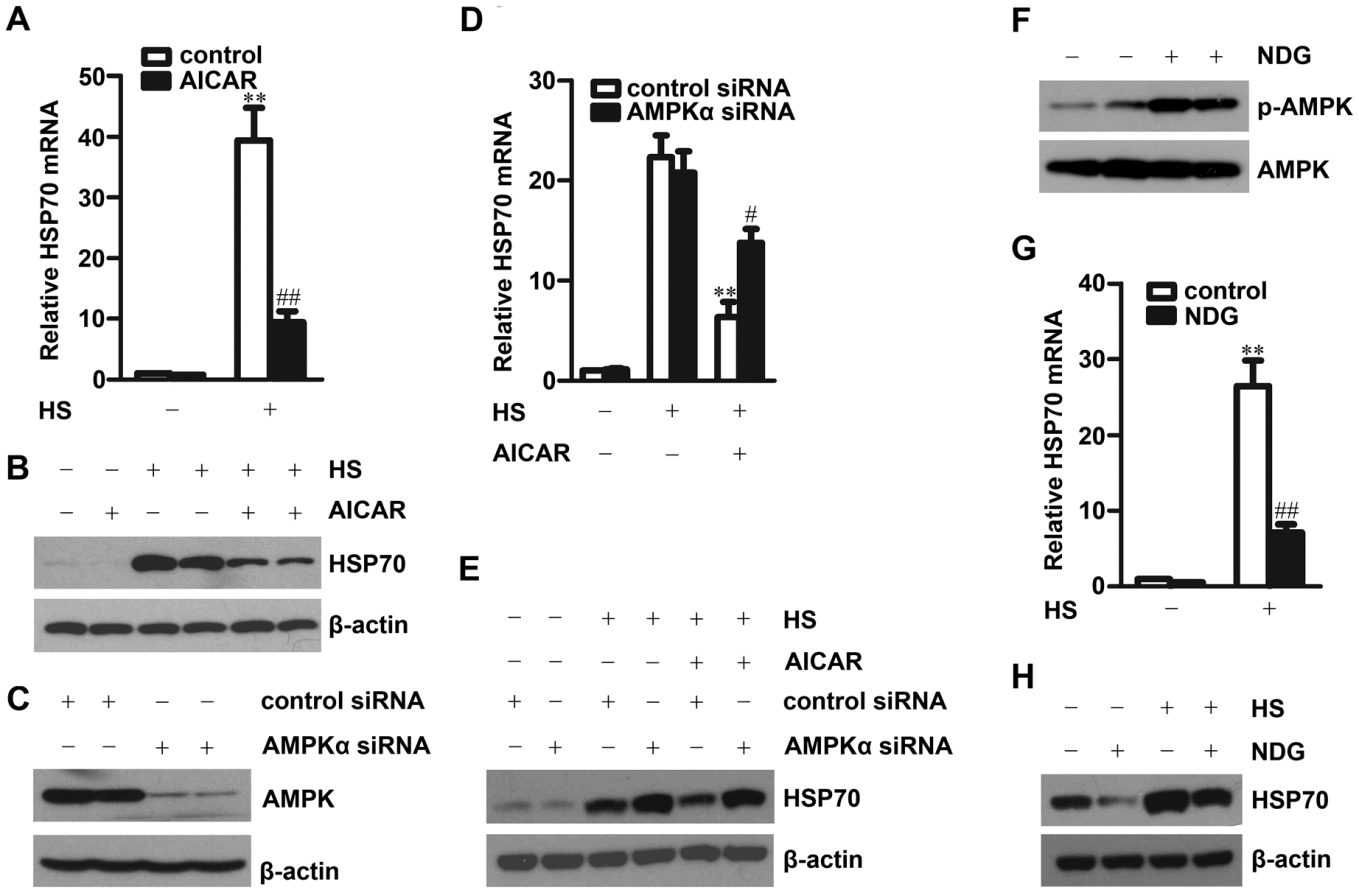
Activation of AMPK inhibits HSP70 expression in response to heat stress. **A–B.** HepG2 cells pretreated with or without 1 mM AICAR for 15 min were exposed to 42°C (HS) for 1 h, HSP70 expression was examined by real-time PCR (**A**) and Western blot after recovery at 37°C for 5 h (**B**). mean ± SEM, n = 3. **P<0.01 vs. cells without heat stress; ^##^P<0.01 vs. cells cultured in control medium under heat stress. **C–E.** HepG2 cells transfected with AMPKα siRNA or control siRNA for 48 h were examined for AMPKα protein level by Western blot (**C**), or treated with or without 1 mM AICAR for 15 min followed by exposure to 42°C for 1 h, then examined HSP70 expression by real-time PCR (**D**) and Western blot after recovery at 37°C for 5 h (**E**). mean ± SEM, n = 3. **P<0.01 vs. control siRNA transfected cells without AICAR treatment under heat stress; ^#^P<0.05, vs. AMPKα siRNA transfected cells without AICAR treatment under heat stress. **F.** HepG2 cells were treated with 2 mM NaN_3_ combined with 50 mM 2-Deoxyglucose (NDG) for 30 min, then examined for AMPKα phosphorylation by Western blot. **G–H.** HepG2 cells pretreated with or without NDG for 30 min were exposed to 42°C for 1 h, HSP70 mRNA was examined by real-time PCR (**G**), HSP70 protein level was examined by Western blot after recovery at 37°C for 5 h (**H**). mean ± SEM, n = 3. **P<0.01 vs. cells without heat stress. ^##^P<0.01 vs. cells without NDG treatment under heat stress. **B**, **C**, **E**, **F** and **H**: experiments were performed at least three times and the representative results are shown.

The dramatic induction of heat shock proteins is an unifying and adaptive response that enhances cell survival from stress. HSP70 is one of the most highly induced heat shock proteins in response to heat stress. So we examined the relationship between AMPK inhibition and HSP70 upregulation under heat stress. As shown in Figure 2A and B, heat stress significantly induced HSP70 expression at both mRNA and protein levels in HepG2 cells. Pretreatment of the cells with AICAR significantly inhibited heat-induced HSP70 expression. Similar results were obtained from nonspecific siRNA transfected HepG2 cells (Figure 2D and E). When HepG2 cells were transfected with AMPKα1/2 specific siRNA to inhibit AMPKα expression (Figure 2C), the inhibitory effect of AICAR on heat stress-induced HSP70 expression was partially reversed at mRNA level and totally reversed at protein level (Figure 2D and E). These results demonstrated that activation of AMPK by AICAR significantly inhibited HSP70 expression in response to heat stress, which suggests that inhibition of AMPK under heat stress contributes to HSP70 upregulation.

AMPK, as a cellular energy sensor, is activated by the reduction of cellular ATP levels. To get more evidence for negative regulation of HSP70 expression by AMPK under heat stress, we used NaN_3_ combined with 2-DG (2-deoxyglucose) to activate AMPK. Treatment of HepG2 cells with these two compounds significantly induced AMPK phosphorylation (Figure 2F) and inhibited heat stress-induced HSP70 expression at both mRNA and protein levels (Figure 2G and H). These results further support that HSP70 is negatively regulated by AMPK under heat stress.

**Figure 3:**
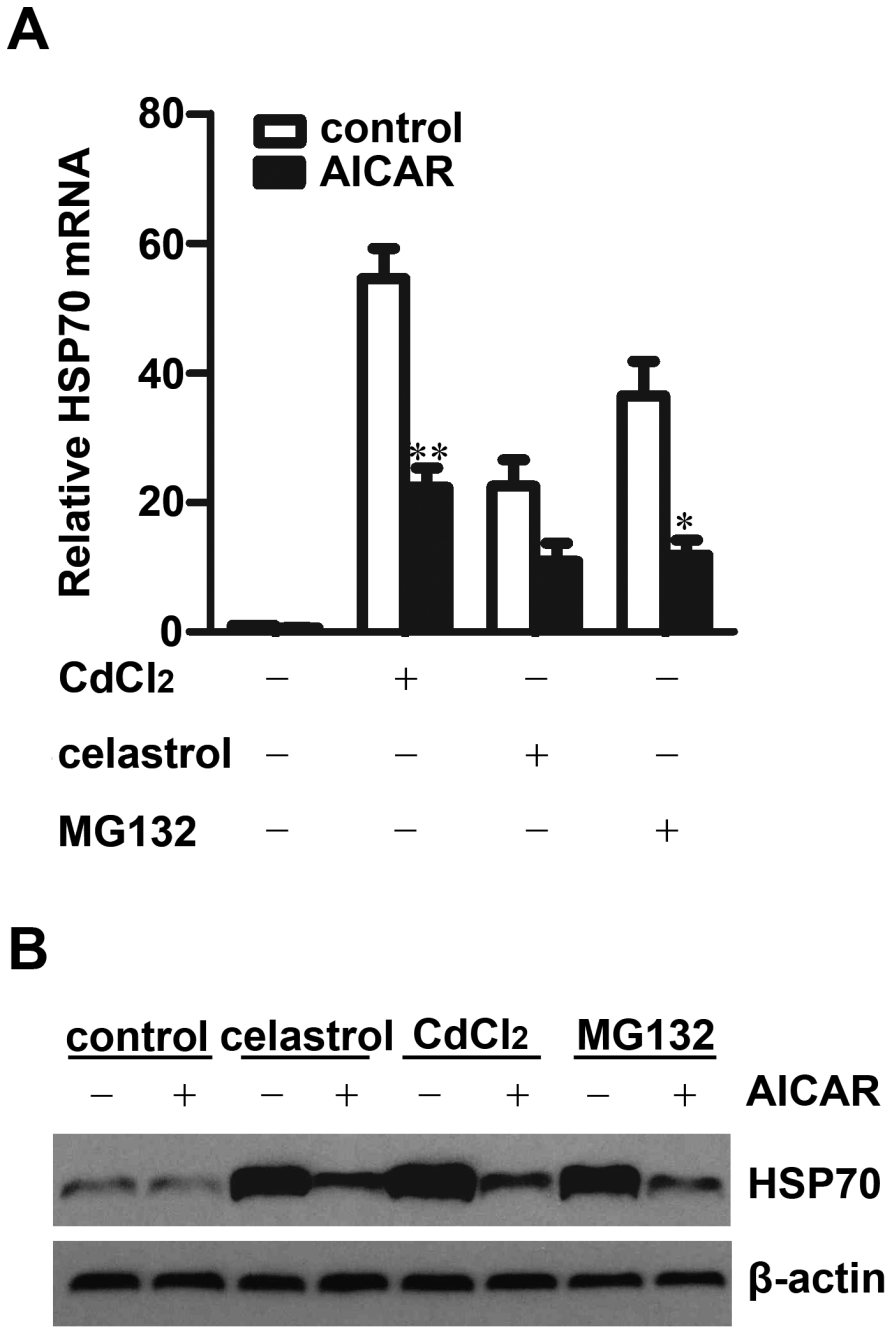
Activation of AMPK inhibits HSP70 expression in response to various stresses. HepG2 cells pretreated with or without 1 mM AICAR for 15 min were incubated with 5 µM celastrol for 1 h, 50 µM CdCl_2_, or 20 µM MG132 for 5 h, HSP70 mRNA levels were examined by real-time PCR (**A**). HSP70 protein levels were examined by Western blot after incubation with CdCl_2_ or MG132 for another 3 h, or replacing celastrol with fresh medium for 5 h (**B**). mean ± SEM, n = 3. *P<0.05, **P<0.01 vs.\ stressor stimulated cells without AICAR pretreatment. **B:** experiments were performed at least three times and the representative results are shown.

We also checked the involvement of AMPK in HSP70 upregulation in response to other stressors which could induce HSP expression. As shown in Figure 3A and B, stimulation of HepG2 cells with celastrol, CdCl_2_, or MG132, significantly induced HSP70 expression at mRNA and protein levels. Pretreatment with AICAR suppressed HSP70 expression induced by these stressors. These results suggest that the negative regulation of HSP70 expression by AMPK might be a common event under stress.

### Effects of AMPK activation on HSF1 activity and HSP70 mRNA stability under heat stress

**Figure 4:**
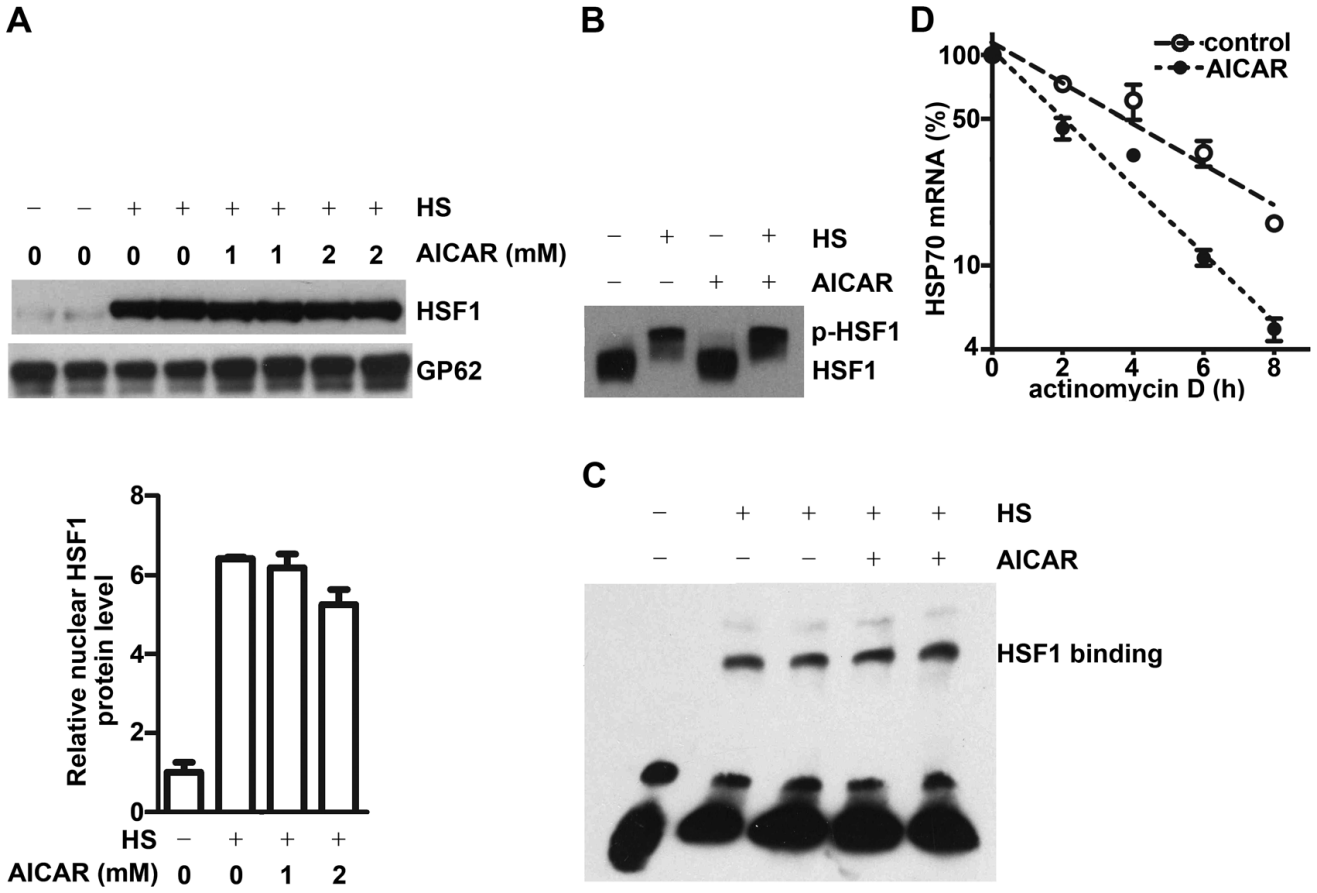
Effects of AMPK activation on HSF1 activity and HSP70 mRNA stability under heat stress. **A–C.** HepG2 cells pretreated with or without different concentrations of AICAR for 15 min were exposed to 42°C for 30 min, then examined for nuclear HSF1 protein level (**A**) and HSF1 phosphorylation (**B**) by Western blot, or HSF1/HSE binding by EMSA (**C**). A: mean ± SEM, n = 3. Representative gels were shown. Nuclear pore complex protein gp62 (GP62) was shown for protein loading. **D.** HepG2 cells were exposed to 42°C for 1 h, then treated with 5 µg/ml actinomycin D in the presence or absence of 1 mM AICAR and kept at 42°C for indicated time intervals. HSP70 mRNA levels were examined by real-time PCR. Exponential decay curves of HSP70 mRNA were plotted to calculate the half life of HSP70 mRNA. Mean ± SEM, n = 3. **B–C:** experiments were performed at least three times and the representative results are shown.

Heat shock transcription factor 1 (HSF1) is the major transcription factor controlling heat shock protein gene transcription [6], [7]. To explore the mechanisms underlying HSP70 negative regulation by AMPK under stress, we first examined the effect of AMPK on heat stress-induced HSF1 phosphorylation and nuclear translocation, as well as HSF1 binding with the HSE in HSP70 gene by Western blot and EMSA, respectively. As shown in Figure 4A–C, pretreatment of HepG2 cells with AICAR to activate AMPK had no effect on these events driven by heat stress. We then examined whether AMPK modulate HSP70 expression at post-transcriptional level by checking the effect of AMPK activation on HSP70 mRNA stability under heat stress. We found that after activation of AMPK with AICAR under heat stress, the half-life of HSP70 mRNA was significantly decreased from 3.81±0.32 h in the control cells to 2.00±0.11 h in the AICAR treated cells (P<0.01, n = 3) (Figure 4D), indicating that HSP70 mRNA was significantly stabilized by the AMPK inhibition under heat stress. This stabilization effect, at least in part, contributed to the dramatic increase of HSP70 mRNA under heat stress.

## Discussion

In the present study, we found that heat stress inhibited AMPK activity by phosphatase 2A (PP2A)-mediated AMPK α subunit dephosphorylation, and AMPK inhibition enhanced HSP70 expression. AMPK activation had no effect on heat stress-induced HSF1 activation but significantly decreased HSP70 mRNA stability. These results demonstrate that under heat stress, PP2A mediated AMPK inhibition upregulates HSP70 at least partly through increase of HSP70 mRNA stability.

AMPK is a cellular energy sensor activated by metabolic stresses that either inhibit ATP synthesis or accelerate ATP consumption. Although Corton et al. [8] reported that AMPK activity was increased under heat stress in rat primary hepatocytes, Kodiha et al. [9] found that heat stress inhibited AMPK by dephosphoryltion of AMPKα in both Hela and 293 cells. Our results showed that under heat stress, AMPKα was dephosphorylated not only in human cells (HepG2, 293T), but also in rodent cells (C2C12, Hepa1-6, bEnd3 and MIN6), suggesting that AMPK inhibition is an universal response to heat stress. ACC and PEPCK are two molecules downstream of AMPK. We found that the inhibition of AMPK under heat stress in turn led to the activation of ACC and upregulation of PEPCK, demonstrating the functional inhibition of AMPK under heat stress. We further found that PP2A activation, rather than intracellular ATP alteration, resulted in AMPKα dephosphorylation under heat stress. PP2A has been reported to be activated by ceramide [18], [19]. Recently, Wu et al. [14] reported that cermaide mediated palmitate-induced PP2A activation and subsequent AMPK inhibition. As intracellular ceramide rapidly increases upon heat stress [20], [21], heat stress-induced PP2A activation may be also mediated by ceramide but needs further investigation. Although PP2C has also been reported to be capable of inactivating AMPK [12], our results showed that PP2A knock-down by RNAi almost totally reversed the inhibition of AMPK by heat stress, suggesting that PP2A plays major role in AMPK inhibition in response to heat stress.

By using AMPK activator and siRNA targeting AMPKα, we revealed that the inhibition of AMPK under heat stress contributed to HSP70 upregulation. Furthermore, activation of AMPK with AICAR also inhibited HSP70 induction by other HSP inducers, including celastrol, CdCl_2_ and MG132. Therefore, the negative regulation of HSP70 by AMPK may be a common mechanism involved in HSP70 induction under stress.

As an energy sensor, AMPK is a key regulator of catabolic versus anabolic processes. Activated AMPK may modulate key metabolic enzymes via direct phosphorylation, may directly and indirectly regulate transcriptional programs through phosphorylation events. How AMPK activation regulates the expression of a number of genes involved in metabolism is only beginning to be elucidated [22]. The mechanisms underlying gene regulation by AMPK inhibition remains unclear. Our results showed that under heat stress, treatment of cell with AMPK activator AICAR had no effect on HSF1 activation but reduced the HSP70 mRNA stability, suggesting that AMPK inhibition under heat stress enhances HSP70 expression at least partially through the increase of HSP70 mRNA stability. Metabolic stress induced AMPK activation can inhibit protein synthesis through two pathways: inhibition of the elongation step of translation through activation of elongation factor-2 kinase, and inhibition of the initiation step for protein synthesis through inhibiting target-of-rapamycin (TOR) pathway [1]. It's possible that the inactivation of AMPK could enhance protein synthesis. Inactivation of AMPK under heat stress may contribute to HSP70 protein synthesis but needs further investigation.

Besides the metabolic activity of AMPK, there is growing evidence that AMPK activation plays negative roles in cell survival and growth [3], [4], [23]. HSPs are critical components of defense mechanism against injury associated with adverse stresses [24]. Our results for the first time demonstrated the involvement of AMPK in modulation of heat shock protein gene expression which is a novel mechanism of heat shock response. Inhibition of AMPK under heat stress and the link between AMPK and HSP may be beneficial to cell survival.

## Materials and Methods

### Cell culture and treatment

All cells were cultured in DMEM containing 10% FBS and antibiotics. Short-time heat stress (≤1 h) was performed by submersion of cells in a pre-warmed circulating water bath at 42°C. For long-time heat shock stress, the cells were incubated at 42°C in a humidified 5% CO_2_ atmosphere incubator. AICAR was purchased from Cell Signaling Technology (New England Biolabs, Beverly, MA). CdCl_2_, sodium azide (NaN_3_) and 2-Deoxyglucose were from Sigma (Louis, MO). Celastrol, MG132 and actinomycin D were from Calbiochem (Darmstadt, Germany).

### siRNA transfection

bEnd.3 or HepG2 [25] cells were transfected with siRNA by using lipofectamine 2000 (Invitrogen). The final concentration of siRNA was 30 nM. AMPKα1/2 siRNA was from Santa Cruz Biotechnology (Santa Cruz, CA). The duplexes of siRNA targeting PP2A catalytic-subunit mRNA (targeting sequence 5′-GAATCCAACGTTCAAGAGG-3′) and control siRNA (scrambled sequence) were synthesized by GenePharma (Shanghai, China).

### ATP assay

The intracellular ATP contents were detected by ENLITEN® ATP Assay System Bioluminescence Detection Kit (Promega) according to manufacturer's instructions.

### Real-time PCR

Total RNA was extracted from cells using the Trizol reagent (Invitrogen, Carlsbad, CA, USA) and depleted of contaminating DNA with RNase-free DNase. cDNA was synthesized from 2 µg RNA with M-MuLV reverse transcriptase and random hexamer (Fermentas, Burlington, Ontario, Canada). Reverse-transcribed cDNA in triplicate samples were checked for target mRNA level by quantitative real-time PCR with Power SYBR Green PCR master Mix (Applied Biosystems Inc, Warrington WAI4SR, UK) on ABI Prism 7500 sequence detector (Applied Biosystems Inc, Foster City, CA, USA). Primers used in the experiments were: Human PEPCK: 5′- CATAAAGGCAAAATCATCATGCA (sense), 5′- TTGCCGAAGTTGTAGCCAAA (antisense); Human HSP70: 5′-TGTCGTCCAGCACCCAGGCCAGC (sense), 5′-GCTCTTGTTCAGGTCGCGCCCG (antisense). Human 36B4: 5′-TGCTGAACATGCTCAAC (sense), 5′-GTCGAACACCTGCTGGATGAC (antisense). Amplification of the PEPCK or HSP70 cDNA was normalized to 36B4 expression. Relative levels of target mRNA expression were calculated using the 2^-ΔΔC^_T_ method.

### Immunoblotting

Cell lysate was prepared with cold RIPA lysis buffer as previously reported [26]. Nuclear extract was prepared by NE-PER™ Nuclear and Cytoplasmic Extraction Reagents (Thermo, Rochford, USA) according to the manufacturer's instructions. The protein concentration was determined using Bradford assay. Proteins from cell lysates or nuclear extracts were separated by SDS-PAGE, transferred onto polyvinylidene difluoride (PVDF) membrane (Millipore Corporation, Bedford, MA) and probed with antibodies against HSP70, HSF1, p(T172)-AMPKα, AMPKα, p-ACC, or ACC (Cell Signaling Technology, Beverly, MA), followed by incubation with a horseradish peroxidase-conjugated secondary antibody. Immunoreactive bands were detected by Supersignal West Pico chemiluminescent substrate (Pierce, Rockford, IL, USA) and X-Omat BT film (Eastman Kodak Company, Rochester, New York, USA). The membranes were also probed with antibodies against β-actin or nuclear pore complex protein gp62 (GP62) and corresponding secondary antibodies to ensure equal loading of cytoplasmic protein and nuclear protein, respectively. Immunoblot results were quantified by using Quantity One software with Chemi Doc (Bio-RAD, Segrate, ITALY).

### Electrophoretic Mobility Shift Assay (EMSA)

The nuclear extract of cells was prepared and the protein concentration was determined as described above. EMSA was performed with LightShift Chemiluminescent EMSA kit (Pierce Chemical Co., Rockford, USA) to detect HSF1 DNA binding activity by using biotin-labeled HSE-containing oligonucleotides located in the promoter region of HSP70 gene ((−122)GATCCGGCGAAACCCCTGGAATATTCCCCGACCT (−90)).

### Statistical analysis

Results are expressed as means ± SEM of at least three independent experiments. Statistical analysis was performed using Student's t test.

## References

[1] Hardie DG (2007). AMP-activated/SNF1 protein kinases: conserved guardians of cellular energy.. Nat Rev Mol Cell Biol.

[2] Li C, Keaney JF (2010). AMP-activated protein kinase: a stress-responsive kinase with implications for cardiovascular disease.. Curr Opin Pharmacol.

[3] Meisse D, Van de Casteele M, Beauloye C, Hainault I, Kefas BA (2002). Sustained activation of AMP-activated protein kinase induces c-Jun N-terminal kinase activation and apoptosis in liver cells.. FEBS Lett.

[4] Kefas BA, Heimberg H, Vaulont S, Meisse D, Hue L (2003). AICA-riboside induces apoptosis of pancreatic beta cells through stimulation of AMP-activated protein kinase.. Diabetologia.

[5] Mayer MP, Bukau B (2005). Hsp70 chaperones: cellular functions and molecular mechanism.. Cell Mol Life Sci.

[6] Sistonen L, Sarge KD, Morimoto RI (1994). Human heat shock factors 1 and 2 are differentially activated and can synergistically induce hsp70 gene transcription.. Mol Cell Biol.

[7] Sharma A, Meena AS, Bhat MK (2010). Hyperthermia-associated carboplatin resistance:differential role of p53, HSF1 and Hsp70 in hepatoma cells.. Cancer Sci.

[8] Corton JM, Gillespie JG, Hardie DG (1994). Role of the AMP-activated protein kinase in the cellular stress response.. Curr Biol.

[9] Kodiha M, Rassi JG, Brown CM, Stochaj U (2007). Localization of AMP kinase is regulated by stress, cell density, and signaling through the MEK->ERK1/2 pathway.. Am J Physiol Cell Physiol.

[10] Jung JH, Lee JO, Kim JH, Lee SK, You GY (2010). Quercetin suppresses HeLa cell viability via AMPK-induced HSP70 and EGFR down-regulation.. J Cell Physiol.

[11] Gimeno-Alcañiz JV, Sanz P (2003). Glucose and type 2A protein phosphatase regulate the interaction between catalytic and regulatory subunits of AMP-activated protein kinase.. J Mol Biol.

[12] Davies SP, Helps NR, Cohen PT, Hardie DG (1995). 5′-AMP inhibits dephosphorylation, as well as promoting phosphorylation, of the AMP-activated protein kinase. Studies using bacterially expressed human protein phosphatase-2C alpha and native bovine protein phosphatase-2AC.. FEBS Lett.

[13] Ravnskjaer K, Boergesen M, Dalgaard LT, Mandrup S (2006). Glucose-induced repression of PPARalpha gene expression in pancreatic beta-cells involves PP2A activation and AMPK inactivation.. J Mol Endocrinol.

[14] Wu Y, Song P, Xu J, Zhang M, Zou MH (2007). Activation of protein phosphatase 2A by palmitate inhibits AMP-activated protein kinase.. J Biol Chem.

[15] Liangpunsakul S, Sozio MS, Shin E, Zhao Z, Xu Y (2010). Inhibitory effect of ethanol on AMPK phosphorylation is mediated in part through elevated ceramide levels.. Am J Physiol Gastrointest Liver Physiol.

[16] Bialojan C, Takai A (1988). Inhibitory effect of a marine-sponge toxin, okadaic acid, on protein phosphatases. Specificity and kinetics.. Biochem J.

[17] Favre B, Turowski P, Hemmings BA (1997). Differential inhibition and posttranslational modification of protein phosphatase 1 and 2A in MCF7 cells treated with calyculin-A, okadaic acid, and tautomycin.. J Biol Chem.

[18] Lee JY, Hannun YA, Obeid LM (1996). Ceramide inactivates cellular protein kinase Calpha.. J Biol Chem.

[19] Ruvolo PP, Deng X, Ito T, Carr BK, May WS (1999). Ceramide induces Bcl2 dephosphorylation via a mechanism involving mitochondrial PP2A.. J Biol Chem.

[20] Kondo T, Matsuda T, Kitano T, Takahashi A, Tashima M (2000). Role of c-jun expression increased by heat shock and ceramide activated caspase-3 in HL-60 cell apoptosis. Possible involvement of ceramide in heat shock-induced apoptosis.. J Biol Chem.

[21] Jenkins GM, Cowart LA, Signorelli P, Pettus BJ, Chalfant CE (2002). Acute activation of de novo sphingolipid biosynthesis upon heat shock causes an accumulation of ceramide and subsequent dephosphorylation of SR proteins.. J Biol Chem.

[22] Cantó C, Auwerx J (2010). AMP-activated protein kinase and its downstream transcriptional pathways.. Cell Mol Life Sci.

[23] Wang W, Yang X, López de Silanes I, Carling D, Gorospe M (2003). Increased AMP:ATP ratio and AMP-activated protein kinase activity during cellular senescence linked to reduced HuR function.. J Biol Chem.

[24] Rylander MN, Feng Y, Bass J, Diller KR (2005). Thermally induced injury and heat-shock protein expression in cells and tissues.. Ann N Y Acad Sci.

[25] Lin CL, Huang HC, Lin JK (2007). Theaflavins attenuate hepatic lipid accumulation through activating AMPK in human HepG2 cells.. J Lipid Res.

[26] Wang O, Cai K, Pang S, Wang T, Qi D (2008). Mechanisms of glucose-induced expression of pancreatic-derived factor in pancreatic beta-cells.. Endocrinology.

